# Orchestration of Gut–Liver-Associated Transcription Factors in MAFLD: From Cross-Organ Interactions to Therapeutic Innovation

**DOI:** 10.3390/biomedicines13061422

**Published:** 2025-06-10

**Authors:** Ao Liu, Mengting Huang, Yuwen Xi, Xiaoling Deng, Keshu Xu

**Affiliations:** 1 Department of Gastroenterology, Union Hospital, Tongji Medical College, Huazhong University of Science and Technology, 1277 Jiefang Avenue, Wuhan 430022, China; xhdrla@hust.edu.cn (A.L.); xyw0563@hust.edu.cn (Y.X.); 2 Department of Radiology, Union Hospital, Tongji Medical College, Huazhong University of Science and Technology, 1277 Jiefang Avenue, Wuhan 430022, China; huangmengting968@163.com

**Keywords:** metabolic dysfunction-associated fatty liver disease, transcription factors, metabolism, inflammation, microbiota, molecular mechanisms

## Abstract

Metabolic dysfunction-associated fatty liver disease (MAFLD) represents a global health burden, however, therapeutic advancements remain hindered by incomplete insights on mechanisms and suboptimal clinical interventions. This review focused on the transcription factors (TFs) associated with the gut–liver axis, emphasizing their roles as molecular interpreters of systemic crosstalk in MAFLD. We delineate how TF networks integrate metabolic, immune, and gut microbial signals to manage hepatic steatosis, inflammation, and fibrosis. For instance, metabolic TFs such as peroxisome proliferator-activated receptor α (PPARα) and farnesoid X receptor (FXR) are responsible for regulating lipid oxidation and bile acid homeostasis, while immune-related TFs like signal transducer and activator of transcription 3 (STAT3) modulate inflammatory cascades involving immune cells. Emerging evidence highlights microbiota-responsive TFs, like hypoxia-inducible factor 2α (HIF2α) and aryl hydrocarbon receptor (AHR), linking microbial metabolite signaling to hepatic metabolic reprogramming. Critically, TF-centric therapeutic strategies, including selective TF-agonists, small molecules targeted to degrade TF, and microbiota modulation, hold considerable promise for treating MAFLD. By synthesizing these insights, this review underscores the necessity to dissect TF-mediated interorgan communication and proposes a roadmap for translating mechanism discoveries into precision therapies. Future research should prioritize the use of multi-omics approaches to map TF interactions and validate their clinical relevance to MAFLD.

## 1. Introduction

Metabolic dysfunction-associated fatty liver disease (MAFLD) has emerged as a leading cause of chronic liver disease and affects approximately 30% adults worldwide, with its prevalence rising in parallel with obesity and type 2 diabetes [1,2]. Current therapies, including lifestyle modifications and conventional medications, exhibit constrained therapeutic outcomes due to suboptimal patient compliance and side effects [3,4]. These highlight the imperative to elucidate the molecular mechanisms governing pathophysiological abnormalities of MAFLD, particularly those integrating systemic metabolic crosstalk.

The gut–liver axis constitutes a critical regulatory interface in MAFLD progression, characterized by dynamic interactions between the gut microbial metabolites, intestinal barrier integrity, and hepatic metabolic reprogramming. The pathological overproliferation of *Clostridium* spp. drives the excessive accumulation of deoxycholic acid (DCA) through upregulated 7α-dehydroxylase activity, thereby exacerbating hepatocellular injury [5]. Conversely, the microbial metabolites acetate and hyodeoxycholic acid translocate to the liver via the gut–liver axis, where they respectively suppress transcription factor (TF) signal transducer and activator of transcription 3 (STAT3) and activate TF peroxisome proliferator-activated receptor α (PPARα), ameliorating the progression of MAFLD [6,7]. These findings validate the axis as a therapeutic nexus and also underscore the necessity of exploring TFs that mediate the bidirectional signaling.

TFs are DNA-binding proteins that regulate gene expression by recruiting co-activators or repressors. In MAFLD, TFs act as molecular switches, translating extracellular cues (e.g., nutrients, microbial metabolites) into transcriptional programs governing lipid metabolism, immune responses, and fibrogenesis [8,9]. TFs also serve as pivotal molecular mediators in systemic crosstalk due to their capacity to decode and integrate signals from diverse organs including the gut and liver. Despite extensive reviews on MAFLD pathogenesis, comprehensive analyses of TF networks orchestrating gut–liver interactions remain sparse. Existing literature has predominantly focused on isolated pathways (e.g., lipid metabolism or inflammation), overlooking the integrative roles of TFs in synchronizing metabolic, immune, and microbial signals. For instance, in MAFLD, TFs such as PPARα and farnesoid X receptor (FXR) are classical molecular integrators of gut–liver axis signaling. PPARα activation orchestrates fatty acid oxidation (FAO) to counteract lipid accumulation, while FXR coordinates bile acid (BA) homeostasis through enterohepatic circulation and suppresses inflammation via modulation of the intestinal barrier [10,11,12]. A systematic search of the PubMed/Scopus databases (2020–2025) was conducted using the keywords MAFLD, NAFLD, transcription factor, gut–liver axis, metabolism, lipogenesis, steatosis, inflammation, fibrosis, immune signaling, microbiota, therapeutics, some specific metabolites, and TFs (e.g., PPAR, FXR). The inclusion criteria prioritized experimental studies (in vitro, in vivo) and clinical trials directly linking TFs to MAFLD pathogenesis or therapy. This review systematically delineated the molecular mechanisms through which gut–liver-associated TFs mediate metabolic adaptation, inflammation, and the intestinal microenvironment of MAFLD. By elucidating their roles as molecular interpreters of interorgan crosstalk, this article provides a conceptual framework for the development of TF-targeted therapies to restore homeostasis in MAFLD.

## 2. Metabolism-Related TFs in MAFLD

**Figure 1:**
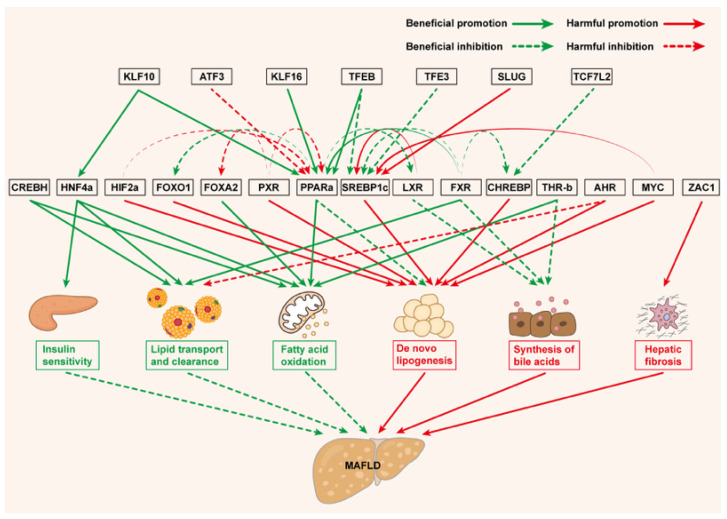
Metabolism-related TFs in MAFLD. Green text suggests beneficial effects on MAFLD, while red text suggests harmful effects on MAFLD.

MAFLD is characterized by multifaceted metabolic disturbances including dysregulated lipid accumulation and impaired glucose homeostasis [13,14]. Central to these processes are metabolism-associated TFs, which orchestrate gene networks governing lipogenesis, lipid oxidation, and BA synthesis. Understanding these TFs provides critical insights into therapeutic strategies targeting metabolic reprogramming in MAFLD (Figure 1).

### 2.1. PPARs

PPARs, including PPARα, PPARβ/δ, and PPARγ, bind to peroxisome proliferator response elements in the promoter regions of target genes and are activated by unsaturated fatty acids and derivatives from the diet, lipogenesis, or lipolysis. PPARα is expressed mainly in the liver and regulates FAO and lipoprotein metabolism. In MAFLD, PPARα suppresses hepatic steatosis by upregulating genes involved in β-oxidation (*CPT1A*, carnitine palmitoyltransferase 1A; *ACOX1*, acyl-CoA oxidase 1) and inhibiting de novo lipogenesis (DNL) (Table 1) [15]. PPARα activation also reduces lipid uptake and very low-density lipoprotein (VLDL) secretion via the transcriptional regulation of CD36 and microsomal triglyceride transfer protein (MTTP) [16]. Hepatic PPARα-deficient mice have aggravated liver steatosis and inflammation, with hyperlipidemia [17]. Similarly, the nuclear factor of activated T-cells c4 inhibition attenuated metabolic dysfunction-associated steatohepatitis (MASH) by relieving PPARα suppression, enhancing FAO [18].

Notably, PPARα interacts with other TFs, further influencing lipid metabolism. In methionine-choline-deficient (MCD) diet-induced MAFLD, PPARα activation reduced lipid accumulation and inflammation via the AMP-activated protein kinase (AMPK)-mediated inhibition of mechanistic target of rapamycin (mTOR)/sterol regulatory element binding protein-1c (SREBP1c) signaling. Meanwhile, Krüppel-like factor 10 (KLF10) activates PPARα, improving glucose tolerance and reducing liver triglycerides (TGs). KLF16 enhances FAO by directly binding to the PPARα promoter, and its overexpression activates PPARα-targeted genes, improving mitochondrial function and insulin sensitivity [49]. Transcription factor EB (TFEB) upregulates PPARα to promote FAO [50]. Activating transcription factor 3 (ATF3) promotes lipogenesis under metabolic stress and worsens lipid accumulation and fibrosis via PPARα inhibition [51].

PPARα agonists enhance fibroblast growth factor 21 (FGF21) secretion, which promotes adipose tissue browning and systemic lipid oxidation [52]. PPARα agonists (pemafibrate and elafibranor) also improve insulin sensitivity, lower TGs, and reduce hepatic inflammation and fibrosis in preclinical models [53,54]. However, clinical trials with PPARα agonists show mixed efficacy, necessitating selective modulators to optimize therapeutic outcomes [53].

PPARβ/δ is widely expressed in the body and enhances FAO and energy uncoupling. Its expression is reduced in the livers of MAFLD patients [55]. Studies in PPARβ/δ-deficient mice showed increased fat accumulation, while transgenic mice had enhanced FAO in adipose tissue [56]. PPARδ also lowers the apoC-III and VLDL receptors, and increases apoA-II [20]. Specific agonists such as GW501516 increased HDL-C and reduced LDL-C and TG in obese monkeys with insulin resistance, suggesting its potential for treating MAFLD [57]. Additionally, PPARβ/δ activation in the liver of mice also prevents the expression of SREBP1c [58].

PPARγ, highly expressed in adipose tissue, regulates adipocyte differentiation, lipid uptake, and energy storage. In preclinical models, PPARγ overexpression exhibits protective effects by reducing hepatic steatosis, inflammatory infiltration, and fibrotic remodeling [59]. PPARγ agonists promote lipid redistribution, facilitating TG mobilization from hepatocytes to adipocytes [21], and reduce blood fatty acids, TG, LDL, and cholesterol by promoting lipid metabolism in adipose tissue [60].

Paradoxically, clinical observations have revealed elevated hepatic PPARγ expression in MASH cases [61,62], and PPARγ-deficient murine models exhibit resistance to diet-induced MASH [22]. This contradiction requires a meticulous dissection of the effects on different cells (hepatocytes and Kupffer cells) and transcriptional crosstalk with parallel regulatory networks.

PPARα/γ agonists like saroglitazar improve insulin resistance, suppress SREBP1c-mediated lipogenesis, and attenuate oxidative stress via nuclear factor erythroid 2 like 2 (NRF2) activation, demonstrating efficacy in preclinical steatosis and fibrosis models [63]. The pan-PPAR agonist lanifibranor reduces hepatic inflammation and fibrosis and improves glucose tolerance, highlighting their therapeutic versatility [64].

### 2.2. SREBP1c

SREBP1c binds to sterol regulatory element 1 in the promoters of sterol biosynthesis-related genes such as low-density lipoprotein receptor (LDLR). Meanwhile, SREBP1c targets genes such as fatty acid synthase (*FASN*), ATP citrate lyase (*ACLY*) and elongation of very long-chain fatty acid-like 6 (*ELOVL6*), thereby transcriptionally activating fatty acid biosynthesis [65].

In MAFLD, insulin resistance and fructose intake activate SREBP1c, increasing the expression of lipogenic enzymes *FASN* and acetyl coenzyme A carboxylase (*ACC*). Overexpression of SREBP1c increases DNL, leading to hepatic steatosis [66]. AMPK activation suppresses SREBP1c, improving insulin sensitivity and lipid oxidation [24,67]. The pharmacological inhibition of SREBP1c (ugonin J or 25-hydroxylanosterol) ameliorates steatosis by suppressing lipid synthesis and enhancing FAO [68,69].

Post-translational modifications modulate SREBP1c activity. Phosphorylation of SREBP1c competes with its ubiquitination, exacerbating hepatic steatosis, whereas the pharmacological inhibition of phosphorylation significantly downregulates SREBP1c expression, ameliorating lipid deposition [70]. In MAFLD, hepatic snail family transcriptional repressor 2 (*Snai2*/*Slug*) epigenetically enhances SREBP1c transcription via histone demethylation, exacerbating lipid accumulation [25].

Notably, crosstalk exists between PPARα and SREBP1c. Under fasting conditions, PPARα promotes FAO to acetyl-CoA and ketone bodies, concomitant with reduced SREBP1c expression. The SREBP1c promoter contains a liver X receptor (LXR) binding site, and PPAR overexpression suppresses SREBP1c via LXR [71]. Conversely, during refed states, SREBP1c-driven lipogenesis is upregulated while PPARα activity declines [72]. In addition, transcription factor E3 (TFE3) antagonizes SREBP1c to reduce DNL by inhibiting its proteolytic activation and chromatin binding [73]. TFEB also suppresses SREBP1c to alleviate hepatic steatosis.

### 2.3. CHREBP

High-carbohydrate diets upregulate carbohydrate response element binding protein (CHREBP), promoting DNL and insulin resistance [74]. CHREBP mediates carbohydrate-induced lipogenesis by activating genes like *FASN*, stearoyl coenzyme A desaturase 1 (*SCD-1*), lipin 1 (*LPIN1*), and *ACLY* [75]. CHREBP activation in hepatocytes elevated serum S100A6, which suppressed mitochondrial respiration and insulin secretion. The pharmacological inhibition of CHREBP/S100A6 could mitigate β-cell dysfunction in MAFLD [76]. Knockdown of CHREBP normalizes DNL-related gene expression (*FASN*, *ACC*) and reduces 40% DNL [77].

Emerging evidence indicates that the hepatic overexpression of CHREBP directly induces hepatic steatosis, whereas the liver-specific suppression of CHREBP in obese murine models attenuates body weight gain, redirects glucose flux toward glycogen synthesis, and mitigates lipid accumulation in the liver [29,78].

CHREBP also interacts with PPARγ to amplify lipid storage, contributing to MAFLD progression [79]. Notably, other TFs have been shown to alter the transcriptional activity of *CHREBP* [80]. Transcription factor 7-like 2 (TCF7L2) suppresses CHREBP O-GlcNAcylation and liver-specific *Tcf7l2* knockout mice exhibit exacerbated steatosis on high-carbohydrate diets due to unrestrained DNL [81].

### 2.4. CREBH

CAMP-responsive element-binding protein H (CREBH) exhibits highly tissue specificity, which is mainly expressed in the liver and is slightly expressed in the small intestine [82]. It binds to both cAMP response elements and box-B-like motifs within the promoter regions of target genes [83].

Under a high-fat diet (HFD), CREBH activation reduces hepatic lipid accumulation and ameliorates histopathological features of steatohepatitis [84]. Mechanistically, CREBH releases a C-terminal fragment that activates lipoprotein lipase. Elevated CREBH-C in obese individuals correlates with improved lipid homeostasis [31]. Furthermore, CREBH directly interacts with the promoter sequences of apoC2-5 and FGF21, enhancing their expression. ApoC2-5 facilitates TG catabolism and clearance [31], while FGF21 promotes FAO and brown adipose tissue thermogenesis [32].

Beyond hepatic functions, intestinal CREBH exerts cholesterol-lowering effects by targeting the promoter of Niemann–Pick C1-like 1 (*NPC1L1*), a key mediator of intestinal cholesterol absorption. CREBH-mediated suppression of *NPC1L1* inhibits dietary cholesterol uptake, enhances intestinal cholesterol efflux, and consequently mitigates hypercholesterolemia [33].

### 2.5. FXR

FXR is highly expressed in both the liver and intestine. Upon binding to BAs such as endogenous chenodeoxycholic acid (CDCA) and cholic acid (CA), FXR stimulates FGF19/FGFR4 signaling to suppress key enzymes in BA biosynthesis including cytochrome P450 family 7 subfamily A member 1 (CYP7A1) and cytochrome P450 family 8 subfamily B member 1 (CYP8B1) [85]. FGF19, a hormone synthesized in ileal enterocytes under the dual activation of BAs and FXR, enters the liver via portal circulation. There, it binds to FGFR4 and other receptors to feedback-inhibit BA synthesis, reducing hepatic lipid overload [86]. FXR agonists such as obeticholic acid, tropifexor, and cilofexor elevate circulating FGF19 levels. Notably, non-steroidal agonists targeting intestinal FXR alter the BA composition (the ratio of CA to taurocholic acid) to inhibit the absorption of polyunsaturated fatty acids and subsequently reduce hepatic TG accumulation [34].

MAFLD patients exhibit gut dysbiosis with increased taurine/glycine-metabolizing bacteria, which elevates secondary BA production. Specifically, elevated levels of FXR-antagonistic DCA and reduced FXR-agonistic CDCA impair FXR signaling. Concomitantly, the serum FGF19 levels are diminished in these patients, while primary and secondary BA concentrations are elevated [87]. FXR knockout mice exhibit elevated serum and hepatic TG, cholesterol, and free FA.

Furthermore, hepatic FXR suppresses the generation of monounsaturated fatty acids [34] and enhances the activity of PPARα, collectively reducing TGs. Additionally, FXR suppresses the transcriptional regulation effects of CHREBP by competitively binding to the promoter region of the key glycolytic enzyme, pyruvate kinase 1 [88]. FXR also induces the small heterodimer partner pathway to inhibit SREBP1c, thus reducing hepatic TG synthesis [89].

Paradoxically, antagonizing intestinal FXR suppresses the ceramide-SREBP1c-mediated DNL, thereby mitigating hepatic lipid accumulation. Discrepancies across studies may arise from variations in experimental models, the impact of interventions on BA pools, and differences in intestinal FXR activity under distinct pathological conditions [90].

### 2.6. PXR

Pregnane X receptor (PXR) shows a crucial endobiotic function in the regulation of lipid metabolism. PXR induces S14, LPIN1, SLC13A5, etc. to promote lipogenesis [91], and targets CD36 and PPARγ to increase lipid accumulation [92]. Constitutively activated PXR mice have hepatic steatosis, and the activation of PXR in human hepatocytes has increased intracellular lipid accumulation [93]. Genetic ablation of *PXR* suppresses c-Jun and LPIN1, leading to enhanced mitochondrial β-oxidation and reduced hepatic lipogenesis [94].

Notably, its activity strongly correlates with the severity of MAFLD [95]. PXR activation by phosphatidylcholine exacerbates MAFLD by upregulating *SLC27A4*, which enhances fatty acid uptake and TG synthesis [36]. *PXR* knockout promotes metabolic reprogramming through the upregulation of PPARα and FGF15 signaling, augmenting energy expenditure while diminishing intestinal lipid absorption [96]. PXR also binds to forkhead box A2 (FOXA2), preventing the binding to its target genes *CPT1a* and 3-hydroxy-3-methylglutaryl-coa synthase 2 (*HMGCS2*), to reduce β-oxidation [97].

### 2.7. LXR

LXR is commonly expressed in various tissues. In hepatic metabolism, LXR has opposite pharmacodynamic effects, promoting lipid deposition and lowering cholesterol. Increased LXR expression promotes the deterioration of MASH by upregulating SREBP1c, FASN, SCD-1, and ACC [37]. The LXR inverse agonist SR9238 reduces hepatic steatosis in the MAFLD model [98].

However, LXR also increases the fecal excretion of BAs by targeting ATP binding cassette subfamily A member 1 (*ABCA1*) and ATP binding cassette subfamily G member 1 (*ABCG1*) [99]. The pharmacological activation of LXR regulates cholesterol homeostasis and increases insulin sensitivity via upregulating *ABCG5/G8* [38].

### 2.8. AHR

Aryl hydrocarbon receptor (AHR) targets multiple key enzymes in lipid metabolism, exacerbating diet-induced obese (DIO) and fibrosis. For instance, AHR binds to dioxin-response elements within the promoter region of the gene *Scd1*, thereby enhancing monounsaturated fatty acid synthesis [100]. Additionally, AHR induces the expression of fatty acid transport-related genes including *CD36*, *LDLR*, *VLDLR*, and *FABP4*, facilitating hepatic fatty acid uptake to exacerbate hepatic lipid overload [101]. Mice with liver-specific *Ahr* overexpression develop spontaneous steatosis and exhibit increased fatty acid intake, CD36 expression, and decreased VLDL-TG secretion [102].

In MAFLD, the AHR inhibitor downregulates the downstream molecules CYP1a1 and TNF-α and reduces oxidative stress and insulin resistance [103]. Notably, whole-body or preadipocyte *Ahr*-deficient mice exhibit complete resistance to hepatic steatosis and DIO [39], whereas this protective effect is not observed in hepatocyte-specific *Ahr* knockout models [104]. These findings suggest that AHR has important cell type-specific functions.

### 2.9. THR-β

Thyroid hormone receptor β (THR-β) is mainly expressed in the liver and binds to thyroid hormone response elements. THR promotes mitochondrial fatty acid uptake and β-oxidation by upregulating CPT1A and medium-chain acyl-coenzyme A dehydrogenase. Notably, THR-β downregulates the expression of SCD-1 and glycerol-3-phosphate acyltransferase-3, which are involved in the hepatic synthesis of TG, while stimulating the expression of adipogenic genes (*FA synthase*, *ACC-α*, *SREBP1c*, and *Carbohydrate-responsive element-binding protein*). The available evidence supports that THR-β-induced hepatic lipolysis is greater than hepatic lipid synthesis. Therefore, FA production does not result in hepatic TG accumulation [105].

Furthermore, THR-β upregulates 3-hydroxy-3-methylglutaryl coenzyme A reductase, increasing hepatic cholesterol synthesis. However, at the same time, it directly recruits to the LDL-R promoter to increase LDL-R expression, clearing cholesterol from the circulation. Overall, THR-β decreases the circulating cholesterol concentrations.

The expression of THR-β in the liver of MASH patients and HFD-fed mice is reduced [106]. THR-β reduces hepatic steatosis and inflammation in both obesity and MASH models. The agonist resmetirom reduces liver weight, hepatic steatosis. and liver enzymes [40].

### 2.10. HNF4α

Hepatocyte nuclear factor 4 α (HNF4α) governs lipid transport by regulating apoB and MTTP, critical for VLDL secretion and HDL metabolism [107]. Meanwhile, HNF4α modulates insulin sensitivity via glucose-6-phosphatase catalytic and phosphoenolpyruvate carboxykinase.

HNF4α expression is reduced in both MAFLD patients and mice models [108]. Its ablation in mice leads to hepatic steatosis and reduced HDL levels [109], while its overexpression has the opposite effects [110]. Additionally, KLF10 enhances lipid oxidation and suppresses lipogenesis by stabilizing HNF4α [111].

### 2.11. FOX

Forkhead box O1 (FOXO1) drives gluconeogenesis and integrates insulin signaling and lipid metabolism. An increased hepatic expression of FOXO1 was found in insulin-resistant livers, accompanied by increased glucose production and fat deposition [112]. Furthermore, hepatic FOXO1 activation by insulin resistance promotes gluconeogenesis and lipogenesis, exacerbating MAFLD. Adipocyte-specific *Foxo1* deletion in mice reduces 5-lipoxygenase expression and leukotriene B4 production, improving systemic insulin sensitivity and attenuating hepatic steatosis [43]. Moreover, increased PPARα expression interferes with the binding of FOXO1 to the target DNA promoter and reduces their expression [113].

FOXA2 regulates mitochondrial FAO by upregulating *CPT2*. In MAFLD, insulin signaling suppresses FOXA2 nuclear translocation, impairing lipid oxidation. The overexpression of family with sequence similarity 3 member A (FAM3A), which activates FOXA2 via CaM-dependent pathways, reduces hepatic TG and endoplasmic reticulum (ER) stress. Imipramine, an antidepressant, enhances FOXA2 activity, ameliorating steatosis in obese mice [44].

### 2.12. HIF2α

Hepatic hypoxia-inducible factor 2α (HIF2α) participates in lipid metabolism by upregulating the fatty acid synthesis genes *sterol regulatory element binding transcription factor 1* and *FASN* as well as fatty acid uptake gene *CD36* while downregulating β-oxidation genes *PPARα* and *ACOX1* [46]. Under normoxic conditions, HIF2α undergoes rapid degradation mediated by prolyl hydroxylase domain enzymes (PHDs). However, under hypoxic conditions, PHD activity is suppressed, allowing for HIF2α stabilization. Hepatic-specific disruption of PHD2 and PHD3 triggers HIF2α overexpression and exacerbates hepatic steatosis [45].

### 2.13. MYC

Myelocytomatosis (MYC) promotes lipogenesis and suppresses FAO. MYC induces SREBP1, and their coordinated activation promotes fatty acid synthesis, driving fatty acid chain elongation from glucose and glutamine-derived carbon sources [114].

Elevated MYC expression is observed in ileal biopsies from individuals with obesity. Intestinal MYC deletion in mice ameliorates DIO, insulin resistance, and hepatic steatosis by enhancing glucagon like peptide 1 (GLP-1) secretion and reducing ceramide [47]. GLP-1, a gut-derived incretin hormone, potentiates glucose-stimulated insulin secretion while suppressing appetite and delaying gastric emptying [115]. Furthermore, MYC directly targets ceramide synthase 4, thereby activating de novo ceramide synthesis. Ceramide significantly upregulates fatty acid uptake and synthesis via the direct modulation of CD36 and SREBP1c [116]. Exogenous ceramide administration exacerbates metabolic dysfunction in murine models [117].

In addition to the TFs above-mentioned, there are also other TFs that have shown potential for targeting MAFLD, although they are currently understudied. For instance, zinc-finger protein regulator of apoptosis and cell-cycle arrest 1 (ZAC1), an imprinted gene network regulator, is upregulated in juvenile MAFLD models. Postnatal ZAC1 overexpression drives hepatic fibrosis via TGF-β1 and collagen type VI α2 activation [48]. Hepatocyte-specific *Zac1* overexpression induces profibrogenic pathways, while its inhibition ameliorates steatosis. ZAC1 represents a potential target for early-life MAFLD prevention.

Collectively, PPARα, SREBP1c, FXR, LXR, etc. form an interdependent regulatory network coordinating hepatic lipid oxidation, bile acid flux, and lipogenesis. Dysregulation in any node disrupts metabolic homeostasis, as evidenced by PPARα downregulation exacerbating steatosis while FXR agonism ameliorates it. However, contradictory outcomes (e.g., LXR effects) highlight context-dependent TF functions. Critical knowledge gaps persist regarding: (1) Compensatory mechanisms among metabolic TFs during MAFLD progression and (2) Organ-specific crosstalk (e.g., adipose-liver PPARγ signaling). Future studies should employ tissue-specific knockout models to resolve these complexities.

## 3. Inflammation and Immune-Related TFs in MAFLD

**Figure 2:**
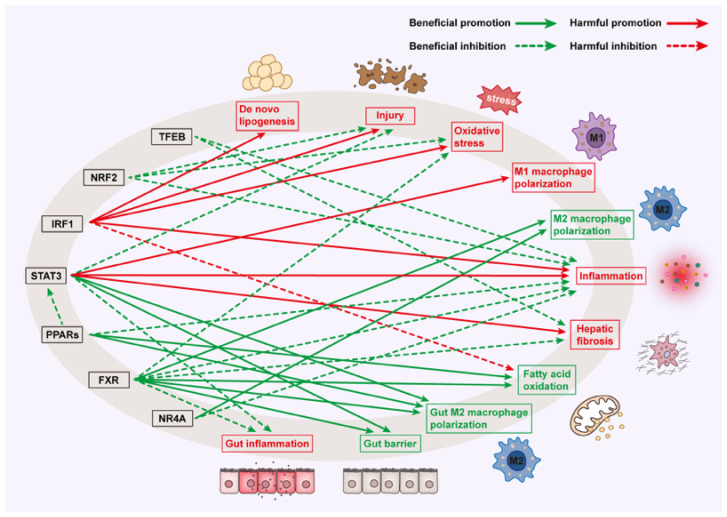
Inflammation and immune-related TFs in MAFLD. Green text suggests beneficial effects on MAFLD, while red text suggests harmful effects on MAFLD.

The immune system is pivotal in maintaining homeostasis and orchestrating regulatory mechanisms of the gut–liver axis. The hepatic microenvironment, where fat accumulates, leads to stress in hepatocytes, activating inflammatory signals and immune cells. In turn, inflammation exacerbates liver injury and metabolic disorders, accelerating MAFLD progression. Notably, in MAFLD, macrophage polarization (M1 pro-inflammatory vs. M2 anti-inflammatory phenotypes) also critically influences gut–liver crosstalk [118]. M1 macrophages exacerbate intestinal permeability via TNF-α, promoting hepatic inflammation, while M2 macrophages mitigate damage through IL-10. STAT3, for instance, regulates this balance by modulating cytokine signaling in Kupffer cells. TFs fulfill a pivotal function in these processes (Figure 2).

### 3.1. PPARs

In MAFLD, PPARα activation ameliorates inflammation in HFD-fed mice by reducing lipid accumulation and oxidative stress (Table 2) [119]. PPARδ enhances mitochondrial function and anti-inflammatory responses and reduces IL-1β, F4/80, and NLR family pyrin domain containing 3 (NLRP3) and enhances M2 macrophage polarization [120]. The PPARδ agonism GW0742 attenuates pro-inflammatory cytokine expression in HepG2 cells and elafibranor reduces fibrosis and inflammation in phase III trials for MASH [121]. Additionally, experimental evidence demonstrates that PPARγ attenuates inflammation through the transcriptional repression of the NF-κB and STAT signaling pathways [122], and its activation in hepatic stellate cells (HSCs) suppresses TGF-β1, with a 58% α-smooth muscle actin (α-SMA) reduction [123].

### 3.2. FXR

In MCD diet-induced MAFLD, FXR activation attenuates oxidative stress and HSC activation by regulating the BA synthesis pathways [126]. Similarly, DWN12088 ameliorated MASH by enhancing FXR-mediated suppression of TGF-β/SMAD family member (Smad)2/3 signaling, thereby reducing HSC activation [135], while hepatocyte-specific *Fxr* knockout exacerbated fibrosis by upregulating TGF-β/Smad3 signaling [136]. FXR also suppresses NLRP3 and NF-κB signaling, reducing pro-inflammatory cytokines (TNF-α, IL-6) and chemokines (monocyte chemoattractant protein-1, MCP-1) in hepatocytes and macrophages, and promotes macrophage polarization toward anti-inflammatory phenotypes [125]. Intestinal FXR activation prevents ileal CD8+ T cell infiltration and improves gut barrier function via G protein-coupled bile acid receptor 1 (GPBAR1)-dependent IL-10 production [137].

### 3.3. STAT3

STAT3 mediates pro-inflammatory and fibrogenic signaling in MAFLD. Transcriptomic analyses of human MAFLD biopsies and CCl_4_-induced fibrosis models have revealed that STAT3 correlates with fibrosis severity [138]. Once activated, STAT3 promotes the transcription of pro-inflammatory (*IL-1β*) and fibrotic genes (*tissue inhibitor of metalloproteinases 1* (*Timp-1*), *α-SMA*, and *zinc finger e-box binding homeobox 2* (*Zeb2*)) [6], while hepatocyte-specific *STAT3* deletion ameliorates fibrosis by suppressing α-SMA and TIMP-1.

Meanwhile, spatial transcriptomic profiling in MASH models revealed activated STAT3 enrichment in hepatic progenitor cells (HPCs), where it sustains HPC expansion and fibrotic progression [138]. STAT3 in Kupffer cells drives IL-17 production, amplifying neutrophil infiltration and fibrosis [128]. IL-17 further upregulates STAT3 phosphorylation in hepatocytes, promoting TGF-β1 secretion and HSC activation [127]. Pharmacological STAT3 inhibitors (napabucasin) ameliorate MASH by suppressing pro-inflammatory macrophage polarization. Targeting STAT3 signaling may disrupt the crosstalk between HSCs and immune cells in MASH.

Notably, there is also evidence confirming that STAT3 acts as an anti-inflammatory signal in MAFLD. The protective effect of IL-22 against diet-induced MAFLD depends on the activation of STAT3 in the intestinal epithelial cells. The therapeutic activation of STAT3 via IL-22 administration improves insulin sensitivity and resolves steatosis and inflammation in diet-induced MAFLD models [129]. IL-6 ameliorates fatty liver in obese mice by promoting STAT3 phosphorylation [139]. Immunosuppressive function of the IL10/STAT3 axis promotes M2 polarization in the ileum [140] and reduces intestinal permeability and subsequent hepatic inflammation [129].

STAT3 also has anti-apoptotic effects. Janus kinase 2/STAT3 signaling in hepatocytes reduced apoptosis by enhancing anti-apoptotic effectors BCL2 like 1 (Bcl-xL) and myeloid cell leukemia 1 (Mcl-1) expression, while STAT3 deficiency exacerbated liver injury [141]. In summary, STAT3 displays complex biological effects in MAFLD. Whether STAT3 activation exacerbates or alleviates disease depends on the cell type in which STAT3 is activated and the model of liver injury.

### 3.4. NRF2

NRF2 mitigates oxidative stress by binding to antioxidant response elements to upregulate detoxifying and antioxidant genes (*Superoxide dismutase 2* (*SOD2*), *Heme oxygenase 1* (*HO-1*), and *NAD(P)H quinone dehydrogenase 1* (*NQO1*)). NRF2 activation via puromycin-sensitive aminopeptidase (PSA) or dimethyl fumarate reduces reactive oxygen species (ROS), IL-6, and TNF-α, and suppresses lipogenic pathways in MAFLD models [130,142,143]. PSA deficiency exacerbates steatosis by impairing NRF2-mediated antioxidant responses, and high-fat diets suppress NRF2 via promoting autophagy impairment and ubiquitination [144].

NRF2 agonists also restore the glutathione levels, counteracting redox imbalance in diet-induced steatosis [145]. In addition, it inhibits gasdermin D expression, reducing hepatocyte pyroptosis and lipid peroxidation [146]. NRF2 enhancers like bardoxolone methyl are under clinical evaluation for MASH due to their anti-inflammatory and antifibrotic effects [147].

### 3.5. IRF1

Interferon regulatory factor 1 (IRF1) exacerbates MAFLD by promoting lipogenesis and oxidative stress. In hepatocytes, IRF1 directly upregulates *Oxysterol binding protein like 3* (*Osbpl3*), *DNA damage inducible transcript 4* (*Ddit4*), and *C-C motif chemokine ligand 2* (*Ccl2*), driving lipid accumulation and inflammation [131]. IRF1 drives macrophage ferroptosis in MASH by binding to the *SLC7A11* promoter, reducing glutathione synthesis by 68%. Meanwhile, IRF1 suppresses AMPK-TFEB-mediated autophagy, impairing lipid clearance and aggravating hepatic steatosis [132]. The inhibition of IRF1 reduces NLRP3 inflammasome activation, collagen deposition, and enhances FAO, suggesting its potential as a therapeutic target.

### 3.6. NR4A

Nuclear receptor subfamily 4 group A (NR4A) regulates intrahepatic T-cell responses and macrophage polarization. In MAFLD, *Nr4a*-deficient mice exhibit exacerbated liver fibrosis due to impaired Kupffer cell survival and enhanced monocyte-derived macrophage recruitment. NR4A deletion in T cells promotes the clonal expansion of pro-inflammatory Th1/Th17 cells but reduces regulatory T-cell activity, worsening hepatic inflammation [133]. NR4A agonists suppress NF-κB, TNF-α, and IL-6 in macrophages, highlighting their potential as anti-inflammatory targets in MASH.

### 3.7. TFEB

TFEB coordinates lysosomal biogenesis and autophagy by regulating genes such as *Lysosomal associated membrane protein 1* (*Lamp1*), *Cathepsin B* (*CTSB*), and *Microtubule associated protein 1 light chain 3 beta* (*Map1lc3b*). Hepatic TFEB activation reduced hepatic TG and NLRP3 expression in CCl_4_-treated mice by promoting autophagic flux [148]. In macrophages, TFEB suppresses NLRP3 inflammasome activation by clearing damaged mitochondria and ROS [144]. TFEB overexpression in murine models restores Kupffer cell efferocytosis, decreasing necroinflammation and fibrosis [134]. Small-molecule TFEB activators, like trehalose, are under investigation for MAFLD therapy.

Collectively, immune-related TFs exhibit intricate cross-regulation in MAFLD inflammation. Unresolved questions include: (1) Temporal hierarchy of TF activation during MASH transition; and (2) Gut microbiome-TF crosstalk in extrahepatic immune priming. Single-cell transcriptomics across disease stages could delineate spatially-resolved inflammatory networks.

## 4. Microbiota and Metabolite-Related TFs in MAFLD

In healthy states, Firmicutes and Bacteroidetes dominate the gut microbiota, producing short-chain fatty acids (SCFAs) that activate anti-inflammatory TFs like AHR. Conversely, Proteobacteria and Fusobacteria are more abundant [118] and alpha diversity is reduced in MAFLD, with elevated ethanol-producing bacteria such as *Klebsiella pneumoniae* and *Limosilactobacillus fermentum* [149]. MAFLD-associated dysbiosis enriches Pseudomonadota and Enterobacteriaceae, generating lipopolysaccharides (LPSs) that activate pro-inflammatory NF-κB, exacerbating hepatic steatosis [150]. Meanwhile, studies in both patients and murine models have consistently demonstrated elevated intestinal permeability in MAFLD cohorts [151,152]. MAFLD patients exhibit a higher prevalence of intestinal bacterial overgrowth compared with healthy controls [153], with metagenomic sequencing revealing an increased abundance of *Escherichia coli* and *Bacteroides vulgatus* [154].

**Figure 3:**
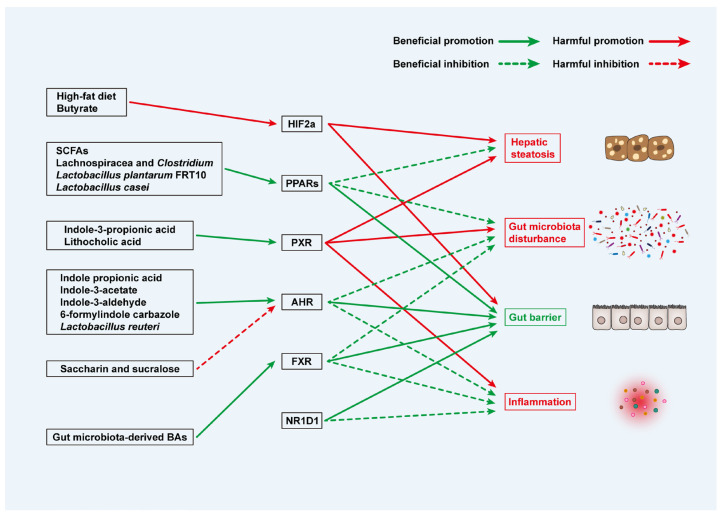
Microbiota and metabolite-related TFs in MAFLD. Green text suggests beneficial effects on MAFLD, while red text suggests harmful effects on MAFLD.

Beyond microbial composition, microbial-derived metabolites significantly influence the gut–liver axis. For example, SCFAs produced by microbiota maintain epithelial barrier integrity. Both human and animal studies have identified reduced intestinal choline bioavailability and the increased portal venous influx of trimethylamine as key features associated with hepatic steatosis (Figure 3).

### 4.1. PPARs

The PPARα/δ agonist restores the expression of tight junction proteins claudin-1 and occludin, thereby ameliorating intestinal integrity and attenuating MASH (Table 3) [140]. Concurrently, the gut microbiota modulates PPAR activity in MASH. Administration of *Lactobacillus casei* enhances hepatic PPARγ activity, which suppresses TLR4 signaling and reduces hepatic steatosis [155]. Similarly, *Lactobacillus plantarum* FRT10 alleviates HFD-induced obesity in mice by activating PPARα [156]. Polysaccharide intervention in MASH models increases the abundance of butyrate-producing bacteria, including Lachnospiraceae and *Clostridium*, while activating the intestinal PPARβ pathway [157].

SCFAs, predominantly acetate synthesized by *Bifidobacterium* and *Lactobacillus*, regulate intestinal pH, promote beneficial microbial growth, and inhibit pathogenic colonization. Reduced SCFAs are consistently observed in clinical MAFLD cohorts and experimental models. SCFAs activate PPARγ, which prevents hepatic lipid accumulation via the uncoupling protein 2-AMPK pathway [164]. Supplementation with α-cyclodextrin elevates SCFA-producing bacteria (*Paenibacillus*, *Bifidobacterium*, and *Lactobacillus*) and upregulates PPARβ/γ expression, modulating adipocyte differentiation and energy expenditure to improve MASH pathology [158].

### 4.2. FXR

Gut microbiota-derived BAs, such as CDCA, activate intestinal FXR, triggering FGF15/19 secretion, which suppresses hepatic BA synthesis via hepatic FGFR4 signaling [165].

In addition, intestinal FXR enhances the expression of intestinal tight junction proteins and regulates microbiota composition [166]. In MASH patients, upregulated markers of gut–vascular barrier (GVB) leakage are observed in the colon, where intestinal FXR ameliorates disrupted GVB through the activation of Wnt/β-catenin signaling [151]. *Fxr*-deficient mice exhibit exacerbated steatosis and fibrosis due to disrupted BA signaling and increased intestinal permeability [167].

Furthermore, intestinal FXR upregulates the expression of inducible nitric oxide synthase (iNOS), IL-18, and angiopoietin 1, which collectively strengthen antimicrobial defense and confer cytoprotective effects against damage [168,169]. Notably, FXR activation prevents chemically induced intestinal inflammation and significantly reduces goblet cell depletion [159].

### 4.3. PXR

Of particular clinical relevance, PXR exhibits anti-inflammatory effects under baseline conditions and pharmacological activation but paradoxically manifests pro-inflammatory characteristics in pathophysiological states such as MAFLD [160]. Emerging evidence revealed an 87% reduction in *Cyp3a* mRNA expression (target gene of PXR) within the livers of germ-free mice, underscoring the indispensable role of gut microbiota in modulating hepatic PXR signaling [170]. Intriguingly, microbial metabolites, including indole-3-propionic acid and lithocholic acid, serve as intestinal PXR agonists [171].

PXR also modulates the gut microbiota composition by downregulating BA-metabolizing bacterial species. PXR activator statins increase the body weight in mice and elevate the abundance of commensal bacteria from the S24-7 family and DCA [172]. Another PXR activator, polybrominated diphenyl ethers, enhances the populations of *Akkermansia muciniphila* and *Allobaculum* spp., alongside elevating unconjugated secondary BAs [173]. PXR-dependent mechanisms increase the Firmicutes/Bacteroidetes ratio (a hallmark of obesity) and specific pro-inflammatory *Lactobacillus* species while reducing anti-obesity *Allobaculum* and anti-inflammatory *Bifidobacterium* [160].

### 4.4. AHR

Many microbial metabolites are AHR ligands. For instance, indole propionic acid activates AHR to reduce TNF-α, IFN-γ, and IL-1β, relieving intestinal inflammation and preventing bacterial translocation [174]. The tryptophan-derived bacterial metabolite indole-3-acetate activates AHR in intestinal epithelial cells, thereby promoting mucin production [175]. Other AHR agonists, indole-3-aldehyde and 6-formylindole carbazole, protect the intestinal mucosa and inhibit inflammation in mice by promoting cytokines IL-22 and IL-10 [176].

In addition, gut microbes like *Lactobacillus reuteri* produce endogenous AHR ligands to suppress intestinal inflammation [177]. Noncaloric artificial sweeteners saccharin and sucralose decrease microbiota-derived AHR ligands and colonic AHR expression, causing MAFLD in mice [161].

### 4.5. HIF2α

Intestinal biopsies from obese individuals have revealed a positive correlation between HIF2α expression and both the body mass index and hepatic enzymes. Meanwhile hypoxia probes indicate reduced partial pressure of oxygen at intestinal villus tips [162]. Studies suggest that HFD promotes HIF2α activation. This may be attributed to gut bacteria-derived butyrate, which consumes oxygen, though the precise mechanism remains unclear [178].

Intestinal epithelium-specific *Hif2α* knockout significantly ameliorates HFD-induced hepatic steatosis and obesity in mice, accompanied by reduced intestinal and serum ceramide levels. Mechanistic studies have revealed that intestinal HIF2α primarily drives the pathogenesis of MAFLD by activating neuraminidase 3, a key enzyme in the ceramide salvage pathway, thereby increasing ceramide production. Notably, the selective HIF2α inhibitor PT2385 demonstrates both preventive and therapeutic potential against these metabolic dysregulations [179].

The role of HIF2α in intestinal barrier regulation exhibits a dual mechanism. The acute activation of HIF2α upregulates creatine kinase, which stabilizes intestinal tight junctions [180]. In contrast, chronic HIF2α activation induces caveolin-1 expression, leading to reduced occludin and the subsequent disruption of tight junctions [181]. Sustained HIF2α activation in intestinal epithelial cells triggers spontaneous inflammatory responses through the direct transcriptional upregulation of *Tnf-α*.

### 4.6. NR1D1

Nuclear receptor subfamily 1 group D member 1 (NR1D1), a core component of the circadian clock, exhibits reduced intestinal expression in murine MASH models, concomitant with heightened intestinal permeability. Chromatin immunoprecipitation revealed the direct binding of NR1D1 to promoters of tight junction *Claudin 1*, *Claudin 3*, and *Zona occludens 3* in murine colonic tissue. Administration of the NR1D1 agonist SR9009 restored intestinal barrier integrity in both the LPS-treated Caco2 cells and MASH mice. Histopathological analysis demonstrated SR9009-mediated augmentation of goblet cell populations and mucin secretion in the colon. Furthermore, SR9009 ameliorated hepatic steatosis, insulin resistance, and inflammation in MASH mice, potentially due to enhanced intestinal barrier function [163].

In general, the gut–liver axis in MAFLD is characterized by a dynamic feedback loop, where hepatic dysfunction actively influences the gut microbiota composition, creating a reciprocal regulatory mechanism. For instance, MAFLD-associated perturbations in bile acid metabolism, such as increased levels of deoxycholic acid, can disrupt intestinal microbial equilibrium, promoting the expansion of pathobionts (e.g., *Escherichia coli*) and reducing commensal species. Concurrently, altered hepatic secretion of proinflammatory cytokines (e.g., TNF-α, IL-6) impairs intestinal epithelial tight junctions, enhancing gut permeability and facilitating bacterial translocation. This process reinforces liver injury through endotoxemia and secondary inflammation. Conversely, gut microbiota-derived metabolites (e.g., SCFAs) or outer-membrane vesicles can modulate hepatic lipid metabolism and inflammation, underscoring the critical role of this bidirectional axis in MAFLD progression [182].

In the gut–liver axis, the microbiota-sensing TFs described above translate microbial signals into hepatic responses. However, several key aspects remain unknown. These include the interactions between strain-specific microbiota and TFs, the plasticity of TFs when microbiota is manipulated, and the competition among microbial metabolites for TF binding. Integrated metagenomics-TF activity profiling in longitudinal cohorts could establish causal links between microbial shifts and TF reprogramming.

Inter-organ communication beyond the gut–liver axis also warrants significant attention [183]. In the liver–heart axis, myocardial hypertrophy triggers the activation of IRF3 and NF-κB in cardiomyocytes via the cyclic GMP-AMP synthase-stimulator of interferon gene (cGAS-STING) pathway, promoting type 1 IFN and the expression of other proinflammatory factors that exacerbate MASH [184]. Cardiac injury induced the cardiac fibroblasts to secrete periostin, which impaired hepatic lipid homeostasis by downregulating PPARα and enhancing triglyceride accumulation in the primary hepatocytes [185].

The gut–brain axis influences hepatic lipid metabolism via FXR-dependent signaling. Intestinal L-cell-derived glucagon-like peptide-1 and 2 (GLP-1/2) regulated insulin secretion and appetite, thus affecting energy intake and metabolism [186]. GLP1/2 dual agonists upregulate hepatic FXR expression, mimicking the FXR agonist effects to indirectly ameliorate hepatic fibrosis and metabolic dysfunction [187]. Notably, *Akkermansia muciniphila* restores brain metabolic homeostasis to reverse MASH-associated cognitive deficits including impaired spatial working memory and novel object recognition [188]. The autonomic nervous system further modulates gastrointestinal motility and microbial composition, with lateral hypothalamic appetite neurons activated by sweets. Aspartame exposure, for example, downregulates lipocalin and PPAR signaling, linking the gut–brain interactions to MAFLD pathogenesis [189].

The hormonal regulation of MAFLD through TFs is also critical. Thyroid hormone (TH) enhanced TFEB, a key TF for lysosomal biogenesis diminished in MASH mice, thereby restoring lysosomal function. TH also reduced SREBP1c to promote FAO [190]. Insulin upregulated a TF, Snail1, to repress *fatty acid synthase* promoter activity, inhibiting adipogenesis [191]. Additionally, cardiac-secreted atrial natriuretic peptide promotes lipolysis and energy expenditure, establishing cross-organ metabolic crosstalk [192]. Recent studies have highlighted a robust association between MAFLD and colorectal cancer (CRC) incidence, exemplified by a retrospective analysis of 1145 patients with metabolic syndrome [193]. Individuals who developed CRC exhibited significantly higher baseline levels of fasting plasma glucose and non-invasive liver fibrosis scores, with MAFLD diagnosed in 68% of CRC cases versus 43% in the non-CRC controls. The Fibrosis-4 index exhibited a high predictive value for CRC (AUC = 0.74, OR = 6.1). Notably, CRC is recognized as an obesity-related cancer and is driven by hyperinsulinemia and dyslipidemia [194,195]. These findings align with the proposed gut–liver axis mechanism, where MAFLD-driven hepatic inflammation and dysregulated metabolism promote intestinal epithelial dysfunction and microbial dysbiosis. Concurrently, hyperinsulinemia in MAFLD may exacerbate intestinal stem cell hyperproliferation and tumor microenvironment inflammation, creating a reciprocal loop between the metabolic and oncogenic pathways.

## 5. Targeted Therapy Strategies of TFs

Drugs for MAFLD are still limited today, but TF-related research is making increasing progress in elucidating pathogenesis and potential therapeutic targets.

PPAR agonists have demonstrated therapeutic efficacy in MAFLD, although they are accompanied by specific constraints such as variable response rates and tissue-specific effects (Table 4). The PPARα agonists fenofibrate and pemafibrate ameliorate dyslipidemia and reduce hepatic stiffness. While these agents have not demonstrated statistically significant improvements in histopathological features [196,197], the PPARγ agonist pioglitazone improves steatosis, inflammation, and ballooning of the liver. It is particularly effective in MASH and prediabetes patients [198], but the side effects limit its widespread use. To reduce the side effects, saroglitazar, a PPARα/γ agonist, was created and ameliorates liver enzymes, liver fat content, insulin resistance, and dyslipidemia in MASH patients [199]. Compared with single or dual PPAR agonists, the pan-PPAR agonist lanifibranor decreased at least 2 points in the activity part of steatosis, activity, and fibrosis scoring system in a phase 2b trial, but increased the risk of diarrhea, nausea, etc. [200].

Phase 2 study of the FXR agonist cilofexor demonstrated improvements in fat accumulation and fibrosis in MASH patients [210]. Phase 3 clinical trials demonstrated that obeticholic acid improves liver histology in MASH patients but faces challenges due to side effects [35]. Hybrid FXR/PPAR agonists are under exploration for enhanced efficacy [222].

The selective THR-β agonist resmetirom reduced the liver fat content, LDL, and apoB of MASH patients in a 36-week clinical phase 2 study. It also reduced markers of fibrosis formation and promoted cholesterol metabolism [218,219]. Another agonist, VK2809, underwent a phase 2 trial and showed beneficial effects on reducing hypercholesterolemia, LDL, and the hepatic lipid content in MASH patients, suggesting its potential for treatment [220].

The LXR agonists, GW3965 and SR9238, are currently not suitable for clinical treatment due to their pleiotropic effects on different tissues. However, the intestinal-specific LXR agonist GW6340 reduces the side effects, effectively inducing LXR target genes, promoting cholesterol efflux, and without increasing TG production [216].

In addition to the drugs that have entered the clinical research phase, there are also emerging therapeutic targets and regulatory mechanisms. Tribbles homologue 3 (TRIB3) is an ER stress sensor and directly interacts with HNF4α, mediating the ER stress-induced ubiquitination degradation of HNF4α. Cell-penetrating peptides (CPPs) are long peptides that have the ability to enter the cell and promote intracellular effects through self- or delivered bioactive cargoes. Designing CPPs targeting the binding sites of HNF4α and TRIB3 disrupt the interaction, thereby attenuating HNF4α degradation and improving metabolism in MAFLD mice [223]. Similarly, proteolysis-targeting chimeras (PROTACs), which are small molecules designed to degrade TFs, significantly reduce the off-target effects and toxicities.

Other emerging therapeutic strategies targeting the gut–liver axis include prebiotics, probiotics, and fecal microbiota transplantation (FMT). Prebiotics—non-digestible food ingredients fermented by gut microbiota to confer host benefits—primarily encompass fructo-oligosaccharides (FOSs), galacto-oligosaccharides, β-glucans, and inulin [118]. Polyphenol extracts or dietary fibers as inulin counteract lipid metabolism dysregulation and liver inflammation in preclinical models [224,225], while the clinical evidence in MAFLD patients remains limited, though FOS supplementation reduced the AST levels in patients [226] and improved the NAS score while increasing *Bifidobacterium* and reducing *Clostridium* clusters [227]. Notably, 2′-fucosyllactose attenuated HFD-induced obesity and glucose intolerance, potentially via upregulation of the transcription factor Hes1, which maintains intestinal stem cell stemness and mucin production [228].

Probiotics, defined as live microorganisms conferring health benefits, show preclinical promise. *Bacteroides thetaiotaomicron* reduced adiposity in HFD-fed mice by modulating the Firmicutes/Bacteroidetes ratio, elevating hepatic folate, and polyunsaturated fatty acids [229]. Meta-analyses indicate that *Lactobacillus* + *Bifidobacterium* + *Streptococcus* combinations may optimally improve liver enzymes, total cholesterol, and TNFα [230]. Nevertheless, some clinical trials have reported negligible effects in MASH patients [231], suggesting that outcomes depend on the probiotic strain, treatment duration, and patient heterogeneity.

FMT—transferring processed stool from healthy donors to restore microbial balance—represents another therapeutic avenue. In HFD-fed mice, FMT elevated beneficial bacteria, enhanced intestinal tight junction protein ZO1 expression, reduced hepatic lipid accumulation, and lowered the NAS scores [232]. Conversely, mice receiving microbiota from MAFLD patients exhibited increased weight, LDL, and liver lipids, alongside elevated hepatic PPARγ and reduced LXR expression [233]. Emerging small-scale clinical trials have demonstrated FMT’s potential to improve lipid metabolism and the gut microbiota composition in MAFLD [234,235], though larger longitudinal studies are warranted to validate efficacy.

A multi-target combinatorial approach based on microbes and metabolites to modulate the activity of TFs may improve the intervention efficacy. For instance, a symbiotic composed of *Lactobacillus acidophilus*, *Bifidobacterium infantis*, and konjac glucomannan oligosaccharides attenuated hepatic steatosis and gut microbiota dysbiosis in HFD-fed mice. It reduces LPS, which penetrates the intestinal barrier and enters the circulation, thereby inhibiting the TLR4/NF-κB pathway to alleviate MAFLD [236].

## 6. Challenges and Future Directions

Challenges persist in MAFLD therapeutics, with current research predominantly relying on animal models and lacking clinical validation. Interspecies metabolic disparities necessitate a rigorous evaluation of therapeutic candidates. Developing human organoid systems to replicate MAFLD microenvironments enables a better assessment of drug efficacy.

Current single-target strategies remain inadequate due to TF network redundancies and shared pathways. Better elucidation and utilization of gut–liver TF crosstalk requires multi-omics integration (metagenomics, metabolomics, single-cell RNA-Seq, etc.) to map the dynamic interorgan regulatory networks, thus enabling coordinated multi-cellular therapeutic approaches.

Finally, future studies should prioritize drug repositioning strategies combined with in silico approaches to accelerate therapeutic discovery. Given the substantial time and resources required for novel drug development, computational methods, including molecular docking, machine learning, and network pharmacology, can efficiently identify existing drugs with potential efficacy against MAFLD. By targeting key regulators of the gut–liver axis (e.g., PPARs, LXRs), this approach may rapidly yield repurposable candidates.

In summary, MAFLD pathogenesis, involving metabolic dysregulation, inflammation, and microbiota-metabolite alterations, are all linked to TFs. Targeting TF modulation enables the precise correction of pathophysiological abnormalities of MAFLD and better treatments for patients.

## Figures and Tables

**Table 1:** Characteristics and associations of metabolism-related TFs in MAFLD.

Transcription Factor	Mechanism	Core Functions	Role in MAFLD
PPARα	Activates β-oxidation genes (*CPT1A*, *ACOX1*); inhibits de novo lipogenesis; reduces lipid uptake and VLDL secretion via transcriptional regulation of *CD36* and *MTTP*; antagonizes *SREBP1c* transcription	Liver: Lipid oxidation Gut: Modulates intestinal lipid absorption	Attenuates MAFLD: Attenuates steatosis via enhanced fatty acid utilization [18,19]
PPARβ/δ	Lowers apoC-III and VLDL receptor and increases apoA-II	Liver: Enhances fatty acid oxidation and energy uncoupling	Attenuates MAFLD: Ameliorates hepatic steatosis [20]
PPARγ	Regulates adipocyte differentiation, lipid uptake and energy storage	Liver: Promote lipid redistribution	Attenuates MAFLD: Improves lipid disorders [21] Exacerbates MAFLD: PPARγ-deficient exhibits resistance to diet-induced MASH [22]
SREBP1c	Drives *ACLY*, *FASN*, and *ELOVL6* expression; phosphorylation of SREBP1c increases its expression	Liver: Lipogenesis and cholesterol biosynthesis	Exacerbates MAFLD: Exacerbates hepatic lipid accumulation [23,24,25]
CHREBP	Upregulates lipogenic genes (*FASN*, *SCD-1*, *LPIN1* and *ACLY*); interacts with PPARγ to amplify lipid storage	Liver: Carbohydrate-to-lipid conversion Gut: Regulates dietary sugar metabolism	Exacerbates MAFLD: Promotes DNL under high-carbohydrate diets [26,27,28]; Induces hepatic steatosis [29,30]
CREBH	Cleaved C-terminal fragment activates lipoprotein lipase; induces apoC2-5 and FGF21; suppresses Niemann-Pick C1-like 1	Liver: Promotes fatty acid oxidation Gut: Inhibits dietary cholesterol uptake; enhances intestinal cholesterol efflux	Attenuates MAFLD: Reduces HFD-induced steatosis [31,32]; mitigates hypercholesterolemia [33]
FXR	Stimulates FGF19/FGFR4 signaling to inhibit bile acid synthase CYP7A1 and CYP8B1; alters the ratio of cholic acid to taurocholic acid	Liver: Inhibits bile acid production; suppresses the generation of monounsaturated fatty acids; Gut: Inhibits the absorption of polyunsaturated fatty acids	Attenuates MAFLD: Reduces hepatic TG accumulation [34]; improves fibrosis in MASH patients [35]
PXR	Targets *CD36* and *PPARγ*; Induces S14, *LPIN1* and *SLC13A5* to promote lipogenesis; upregulates *SLC27A4* to increase fatty acid uptake; activates *c-Jun* and *LPIN1* to decrease mitochondrial β-oxidation; downregulates PPARα, FGF15, and FOXA2 signaling pathways	Liver: Lipid uptake and synthesis	Exacerbates MAFLD: Exacerbates steatosis [36]
LXR	Increases *SREBP1c*, *FASN*, *SCD-1*, and *ACC*; Targets *ABCA1* and *ABCG1*;	Liver: Promotes lipid deposition; Gut: Increases fecal excretion of bile acids	Exacerbates MAFLD: Promotes the deterioration of MASH [37] Attenuates MAFLD: Increases insulin sensitivity [38]
AHR	Promotes *SCD-1* expression to enhance monounsaturated fatty acid synthesis; promotes the expression of fatty acid transport-related genes *CD36*, *LDLR*, *VLDLR*, and *FABP4*;	Liver: Facilitates hepatic fatty acid uptake and accumulation	Exacerbates MAFLD: Promotes hepatic steatosis [39]
THR-β	Upregulates *CPT1A*, medium-chain acyl-coenzyme A dehydrogenase and LDL-R; downregulates *SCD-1* and glycerol-3-phosphate acyltransferase-3; stimulates *FASN*, *ACC-α*, *SREBP1c*, and carbohydrate-responsive element-binding protein;	Liver: Promotes mitochondrial fatty acid uptake and β-oxidation; decreases circulating cholesterol	Attenuates MAFLD: Reduces hepatic steatosis and inflammation [40]
HNF4α	Regulates lipid transport genes *apoB* and *MTTP*, and gluconeogenic genes glucose-6-phosphatase catalytic and phosphoenolpyruvate carboxykinase	Liver: VLDL and HDL secretion; glucose homeostasis	Attenuates MAFLD: Loss promotes steatosis; antagonism reduces VLDL output [41,42]
FOXO1	Induces insulin resistance and gluconeogenesis; regulates autophagy and glycophagy	Liver: Glucose production	Exacerbates MAFLD: Exacerbates MAFLD progression; links adipocyte dysfunction to hepatic insulin resistance [43]
FOXA2	Upregulates *CPT2*; regulated by insulin and CaM signaling	Liver: Enhances mitochondrial oxidation	Attenuates MAFLD: Restores lipid catabolism [44]
HIF2α	Upregulates sterol regulatory element binding transcription factor 1, *FASN*, and *CD36*; downregulates *PPARα* and *ACOX1*	Liver: Promotes fatty acid synthesis and uptake; downregulates fatty acid β-oxidation	Exacerbates MAFLD: Exacerbates hepatic steatosis [45,46]
MYC	Induces *SREBP1*; reduces GLP-1 secretion; activates de novo ceramide synthesis	Liver: Drives DNL; Gut: Reduces GLP-1 secretion	Exacerbates MAFLD: Aggravates hepatic ceramide accumulation and hepatic steatosis [47]
ZAC1	Regulates imprinted genes; activates TGF-β1/Collagen Type VI α2	Liver: Drives hepatic fibrosis	Exacerbates MAFLD: Drives juvenile MAFLD fibrosis [48]

PPAR, peroxisome proliferator-activated receptor; *CPT1A*, carnitine palmitoyltransferase 1A; *ACOX1*, acetyl coenzyme A carboxylase; VLDL, very low-density lipoprotein; *MTTP*, microsomal triglyceride transfer protein; SREBP1c, sterol regulatory element binding protein-1c; *ACLY*, ATP citrate lyase; *FASN*, fatty acid synthase; *ELOVL6*, elongation of very long-chain fatty acids-like 6; CHREBP, carbohydrate response element binding protein; *SCD-1*, stearoyl coenzyme A desaturase 1; *LPIN1*, lipin 1; DNL, de novo lipogenesis; CREBH, cAMP-responsive element-binding protein H; apoC, apolipoprotein C; FGF21, fibroblast growth factor 21; HFD, high-fat diet; FXR, farnesoid X receptor; FGF19, fibroblast growth factor 19; FGFR4, fibroblast growth factor receptor 4; CYP7A1, cytochrome P450 family 7 subfamily A member 1; CYP8B1, cytochrome P450 family 8 subfamily B member 1; TG, triglyceride; MASH, metabolic dysfunction-associated steatohepatitis; PXR, pregnane X receptor; *SLC13A5*, solute carrier family 13 member 5; *SLC27A4*, solute carrier family 27 member 4; FOXA2, forkhead box a2; LXR, liver X receptor; ACC, acetyl coenzyme A carboxylase; ABCA1, ATP binding cassette subfamily A member 1; ABCG1, ATP binding cassette subfamily g member 1; AHR, aryl hydrocarbon receptor; *FABP4*, fatty acid binding protein 4; THR-β, thyroid hormone receptor β; HNF4α, hepatocyte nuclear factor 4α; FOXO1, forkhead box o1; HIF2α, hypoxia-inducible factor 2α; MYC, myelocytomatosis; GLP-1, glucagon like peptide 1; ZAC1, zinc-finger protein regulator of apoptosis and cell-cycle arrest 1; MAFLD, metabolic dysfunction-associated fatty liver disease; TGF-β1, transforming growth factor β1.

**Table 2:** Characteristics and associations of inflammation and immune-related TFs in MAFLD.

Transcription Factor	Main Mechanisms	Core Functions	Association with MAFLD
PPARs	PPARα: reduces lipid accumulation and oxidative stress; PPARδ: reduces IL-1β, F4/80, and NLRP3 inflammasome activity; macrophage polarization to M2 phenotype; PPARγ: Transcriptional repression of *NF-κB* and *STAT*; suppresses TGF-β1	Liver: Inhibits inflammation;	Attenuates MAFLD: Improves NAFLD activity score (NAS) [124]; suppresses inflammation and fibrosis [120]
FXR	Inhibits NLRP3 and NF-κB signaling, reducing pro-inflammatory cytokines (TNF-α, IL-6) and chemokines (MCP-1); promotes macrophage M2 polarization; suppresses TGF-β/SMAD2/3 signaling, and reduces HSC activation; prevents ileal CD8+ T cell infiltration; improves gut barrier function via GPBAR1-dependent IL-10 production	Liver: Suppresses inflammation; Gut: Enhances barrier function	Attenuates MAFLD: Attenuates steatohepatitis and fibrosis [86,125,126]
STAT3	Promotes IL-1β, fibrotic genes (*Timp-1*, *α-SMA* and *Zeb2*), TGF-β1, IL-17 secretion, HPC expansion, and HSC activation; improves insulin sensitivity and resolves steatosis and inflammation; promotes M2 polarization; activates anti-apoptotic pathways in hepatocytes	Liver: Promotes pro-inflammatory and fibrogenic signaling; suppresses apoptosis; Gut: Improves insulin sensitivity and intestinal permeability; resolves inflammation	Exacerbates MAFLD: Promotes inflammation, HSC activation and fibrosis [127,128]; Attenuates MAFLD: Reduces hepatocyte and enterocytes death [129]
NRF2	Induces antioxidant genes (*SOD2*, *HO-1* and *NQO1*); restores glutathione levels; inhibits gasdermin D expression	Liver: Reduces ROS and suppresses lipogenic pathways	Attenuates MAFLD: Attenuates oxidative stress and fibrosis [130]
IRF1	Upregulates Osbpl3, Ddit4, and Ccl2; inhibits AMPK-TFEB autophagy; drives macrophage ferroptosis;	Liver: Promotes lipogenesis and oxidative stress;	Exacerbates MAFLD: Aggravates steatosis and inflammation [131,132]
NR4A	Modulates Treg/Th17 balance; inhibits NF-κB, TNF-α and IL-6 in macrophages	Liver: Suppresses Kupffer cell apoptosis	Attenuates MAFLD: Deficiency exacerbates fibrosis and inflammation; agonists reduce inflammation [133]
TFEB	Induces lysosomal/autophagy genes; clears lipid droplets, damaged mitochondria, and ROS	Liver: Enhances lipid degradation and autophagy	Attenuates MAFLD: Restores hepatocyte function; reduces hepatic TG and fibrosis [134]

NLRP3, NLR family pyrin domain containing 3; NAS, NAFLD activity score; STAT, signal transducer and activator of transcription; MCP-1, monocyte chemoattractant protein-1; SMAD2, SMAD family member 2; HSC, hepatic stellate cells; GPBAR1, G protein-coupled bile acid receptor 1; *Timp*-1, tissue inhibitor of metalloproteinases 1; α-SMA, α-smooth muscle actin; *Zeb2*, zinc finger e-box binding homeobox 2; HPC, hepatic progenitor cells; NRF2, nuclear factor erythroid 2 like 2; SOD2, superoxide dismutase 2; HO-1, heme oxygenase 1; NQO1, NAD(P)H quinone dehydrogenase 1; ROS, reactive oxygen species; IRF1, interferon regulatory factor 1; Osbpl3, oxysterol binding protein like 3; Ddit4, DNA damage inducible transcript 4; Ccl2, C-C motif chemokine ligand 2; AMPK, AMP-activated protein kinase; TFEB, transcription factor EB; NR4A, nuclear receptor subfamily 4 group A; Treg, regulatory T cells; Th17, T helper 17 cells.

**Table 3:** Characteristics and associations of microbiota and metabolite-related TFs in MAFLD.

Transcription Factor	Main Mechanisms	Core Functions	Association with MAFLD
PPARs	Restores the expression of tight junction proteins; *Lactobacillus casei*, *Lactobacillus plantarum* FRT10, Lachnospiraceae, Clostridium, and SCFAs enhance PPARs activity	Liver: Reduces hepatic steatosis; Gut: Activated by bacteria; improves intestinal barrier	Attenuates MAFLD: Prevents hepatic steatosis and flora disturbance [158]
FXR	Suppresses hepatic bile acid synthesis; enhances intestinal tight junction; activates Wnt/β-catenin signaling to ameliorate disrupted GVB; upregulates the expression of iNOS, IL-18, and angiopoietin 1	Gut: Improves intestinal barrier; strengthens antimicrobial defense	Attenuates MAFLD: Ameliorates steatosis and fibrosis [159]
PXR	Decreases bile acid-metabolizing bacteria, *Allobaculum*, *Bifidobacterium*; increases *Akkermansia muciniphila*, *Allobaculum* spp., specific pro-inflammatory *Lactobacillus*, and Firmicutes/Bacteroidetes ratio; elevates deoxycholic acid	Gut: Drives microbial dysbiosis and inflammation	Exacerbates MAFLD: Promotes hepatic injury and lipid metabolism disorders in MAFLD [160]
AHR	Activated by indole propionic acid to reduce TNF-α, IFN-γ, IL-1β; promotes mucin production and intestinal homeostasis	Gut: Protects the intestinal mucosa and intestinal homeostasis; inhibits inflammation	Attenuates MAFLD: Decreased AHR ligands and colonic AHR expression is related to MAFLD [161]; suppresses the intestinal inflammation and prevents bacterial translocation
HIF2α	Butyrate consumes oxygen to activate HIF signaling; activates neuraminidase 3 to increase ceramide; acute activation preserves barrier integrity while chronic activation disrupts tight junctions	Gut: Increases intestinal and serum ceramide	Exacerbates MAFLD: Positively correlates with body mass index and hepatic enzymes; HFD promotes HIF2α activation; promotes hepatic steatosis and obesity [162]
NR1D1	Binds to promoters of *Claudin 1*, *Claudin 3*, and *Zona occludens 3*; agonist SR9009 promotes goblet cell populations and mucin secretion	Gut: Restores intestinal barrier integrity	Attenuates MAFLD: Ameliorates hepatic steatosis, insulin resistance, and inflammation in MASH mice [163]

SCFAs, short-chain fatty acids; GVB, gut–vascular barrier; iNOS, inducible nitric oxide synthase; AHR, aryl hydrocarbon receptor; NR1D1, nuclear receptor subfamily 1 group d member 1.

**Table 4:** Targeted therapy strategies of TFs.

Transcription Factor	Drug	Progress	Challenges
PPARα	Fenofibrate	Improved dyslipidemia and inflammation [201]	Minimal effect on insulin sensitivity or liver histology [196]
	Pemafibrate	Decreased MRE-based liver stiffness	Did not decrease liver fat [197]
PPARβ/δ	Seladelpar	Improvement in insulin sensitivity, liver enzymes, and hepatic steatosis	Termination due to worrisome histology results [202]
	Endurobol/GW501516	Increases HDL-C and reduces LDL-C and TG in obese monkeys with insulin resistance [57]	
PPARγ	Pioglitazone	Reduced histological liver fat, inflammation, and fibrosis [203]	Increased risk of fluid retention, edema, congestive heart failure, and bladder cancer [203,204]
	Rosiglitazone	Improve insulin sensitivity [203]	Does not appear to be as effective as pioglitazone in ameliorating MAFLD, and might cause pro-inflammatory changes [203]
PPARα and γ	Saroglitazar	Improved postprandial triglycerides in people with diabetic dyslipidemia [205]; improved ALT and hepatic fat [199]	No improvement in delta change of NAS from baseline to week 24 biopsy [206]
PPARα and δ	Elafibranor	Resolved NASH without fibrosis worsening in patients [207]	Not met the primary endpoint (NASH resolution) in the large phase 3 trial [207]
PPARα, δ and γ	Lanifibranor	Decreased at least 2 points in the activity part of steatosis, activity, fibrosis scoring system of MASH patients [200]; decreased MAFLD in rodents [208]	Increased the risk of diarrhea, nausea, peripheral edema, anemia, and weight gain [200]
SREBP1c	Oltipraz	Decreased liver fat and BMI [209]	No difference in insulin resistance, liver enzymes, lipids, and cytokines
FXR	Cilofexor	Decreased fat accumulation, serum bile acids, and fibrosis in MASH patients [210]	
	Obeticholic acid	Decreased liver enzymes; improved liver histology in MASH patients [35]	No statistically significant effect [211]; side effect: Pruritus [35]
	Tropifexor	Decreased liver fat [212]; ameliorates liver injury, fibrosis, intestinal barrier injury in piglets [213]	
LXR	GW3965	Alters the VLDL-C and LDL-C levels in hamsters and cynomolgus monkeys; inhibits atherosclerosis in mice [214]	Unsuitable for clinical development due to their pleotropic effects
	SR9238	Inhibition of LXR-driven lipogenesis [215]	Decreased liver lipid content and inflammation in mice [215]
	GW6340	Promotes excretion of (3)H-sterol in feces [216]	
	LXR-623	Regulates lipid levels in monkeys [217]	Termination due to unexpected adverse neurological events
THR-β	Resmetirom	Reduced liver fat content, LDL, and apoB of MASH patients [218]	Higher incidence of transient mild diarrhea and nausea [219]
	VK2809	Reduced liver fat content [220]; decreased hepatic mass and TG in mice [221]	

MRE, magnetic resonance elastography; BMI, body mass index.

## Data Availability

No new data were created or analyzed in this study. Data sharing was not applicable to this article.

## Abbreviations

The following abbreviations are used in this manuscript:

α-SMA	α-Smooth muscle actin
ABCA1	ATP binding cassette subfamily A member 1
ABCG1	ATP binding cassette subfamily G member 1
ACC	Acetyl coenzyme A carboxylase
ACLY	ATP citrate lyase
ACOX1	Acyl-CoA oxidase 1
AHR	Aryl hydrocarbon receptor
AMPK	AMP-activated protein kinase
ApoC	Apolipoprotein C
ATF3	Activating transcription factor 3
BA	Bile acid
Bcl-xL	BCL2 like 1
BMI	Body mass index
CA	Cholic acid
Ccl2	C-C motif chemokine ligand 2
CDCA	Chenodeoxycholic acid
cGAS-STING	Cyclic GMP-AMP synthase-stimulator of interferon genes
CHREBP	Carbohydrate response element binding protein
CPP	Cell-penetrating peptides
CPT1A	Carnitine palmitoyltransferase 1A
CRC	Colorectal cancer
CREBH	cAMP-responsive element-binding protein H
CTSB	Cathepsin B
CYP7A1	Cytochrome P450 family 7 subfamily A member 1
CYP8B1	Cytochrome P450 family 8 subfamily B member 1
Ddit4	DNA damage inducible transcript 4
DIO	Diet-induced obese
DNL	De novo lipogenesis
ELOVL6	Elongation of very long-chain fatty acids-like 6
ER	Endoplasmic reticulum
FABP4	Fatty acid binding protein 4
FAM3A	Family with sequence similarity 3 member A
FAO	Fatty acid oxidation
FASN	Fatty acid synthase
FGFR4	Fibroblast growth factor receptor 4
FGF19	Fibroblast growth factor 19
FGF21	Fibroblast growth factor 21
FMT	Fecal microbiota transplantation
FOS	Fructo-oligosaccharides
FOXA2	Forkhead box a2
FOXO1	Forkhead box o1
FXR	Farnesoid X receptor
GLP-1	Glucagon like peptide 1
GPBAR1	G protein-coupled bile acid receptor 1
GVB	Gut-vascular barrier
HFD	High-fat diet
HIF2α	Hypoxia-inducible factor 2α
HMGCS2	3-Hydroxy-3-methylglutaryl-coa synthase 2
HNF4α	Hepatocyte nuclear factor 4α
HO-1	Heme oxygenase 1
HPC	Hepatic progenitor cells
HSC	Hepatic stellate cells
iNOS	inducible nitric oxide synthase
IRF1	Interferon regulatory factor 1
KLF10	Krüppel-like factor 10
LAMP1	Lysosomal associated membrane protein 1
LDLR	Low-density lipoprotein receptor
LPIN1	Lipin 1
LPS	Lipopolysaccharide
LXR	Liver X receptor
MAFLD	Metabolic dysfunction-associated fatty liver disease
MAP1LC3B	Microtubule associated protein 1 light chain 3 beta
MASH	Metabolic dysfunction-associated steatohepatitis
MCD	Methionine-choline-deficient
Mcl-1	Myeloid cell leukemia 1
MCP-1	Monocyte chemoattractant protein-1
MRE	Magnetic resonance elastography
mTOR	Mechanistic target of rapamycin
MTTP	Microsomal triglyceride transfer protein
MYC	Myelocytomatosis
NAS	NAFLD activity score
NLRP3	NLR family pyrin domain containing 3
NPC1L1	Niemann–Pick c1-like 1
NQO1	NAD(P)H quinone dehydrogenase 1
NR1D1	Nuclear receptor subfamily 1 group d member 1
NR4A	Nuclear receptor subfamily 4 group A
NRF2	Nuclear factor erythroid 2 like 2
Osbpl3	Oxysterol binding protein like 3
PHD	Prolyl hydroxylase domain enzymes
PPAR	Peroxisome proliferator-activated receptor
PROTAC	Proteolysis-targeting chimeras
PSA	Puromycin-sensitive aminopeptidase
PXR	Pregnane X receptor
ROS	Reactive oxygen species
SCD-1	Stearoyl coenzyme A desaturase 1
SCFAs	Short-chain fatty acids
SLC13A5	Solute carrier family 13 member 5
SLC27A4	Solute carrier family 27 member 4
SLUG/SNAI2	Snail family transcriptional repressor 2
SMAD2	SMAD family member 2
SOD2	Superoxide dismutase 2
SREBP1c	Sterol regulatory element binding protein-1c
STAT3	Signal transducer and activator of transcription 3
TCF7L2	Transcription factor 7-like 2
TF	Transcription factor
TFE3	Transcription factor E3
TFEB	Transcription factor EB
TG	Triglyceride
TGF-β1	Transforming growth factor β1
TH	Thyroid hormone
THR-β	Thyroid hormone receptor β
Th17	T helper 17 cells
TIMP-1	Tissue inhibitor of metalloproteinases 1
Treg	Regulatory T cells
TRIB3	Tribbles homologue 3
VLDL	Very low-density lipoprotein
ZAC1	Zinc-finger protein regulator of apoptosis and cell-cycle arrest 1
ZEB2	Zinc finger e-box binding homeobox 2
